# Acquired perforating dermatosis associated with uremic pruritus treated with dupilumab monotherapy: A case report

**DOI:** 10.1016/j.jdcr.2026.03.036

**Published:** 2026-03-26

**Authors:** Sara Mubarak, Hayam AlEnezi, Danah I. AlKandari, Al-Sadat Mosbeh, Ghadeer AlAlawi

**Affiliations:** a Department of Dermatology, Adan Hospital, Kuwait City, Kuwait; b Department of Dermatology, Jaber AlAhmad Hospital, Kuwait City, Kuwait; c Dermatology & Dermatopathology, ICDP, Al-Azhar University, Egypt

**Keywords:** acquired reactive perforating collagenosis (ARPC), dupilumab, perforating dermatoses, uremic pruritus

## Introduction

Acquired perforating dermatoses are a group of acquired skin disorders characterized by elimination of collagen or elastic fibers through the epidermis. These include acquired perforating folliculitis, acquired reactive perforating collagenosis (ARPC), Kyrle disease, and elastosis perforans serpiginosa.^1^ ARPC is a reactive condition that typically starts in adulthood and is caused by chronic scratching. It is characterized histopathologically by transepidermal elimination of collagen and clinically by umbilicated papules or nodules with central keratotic plugs.^2^

ARPC is most frequently associated with chronic kidney disease, diabetes mellitus, and liver disease. In patients with chronic kidney disease, uremic pruritus (UP) is thought to drive the itch–scratch cycle, resulting in lesion development and persistence.

The management of ARPC remains challenging. Conventional therapies including emollients, topical, systemic, or intralesional corticosteroids, oral antihistamines, systemic retinoids, doxycycline, allopurinol, and phototherapy, demonstrated limited efficacy.^1^^,^^3^ Dupilumab is a monoclonal antibody that inhibits interleukin-4 (IL-4) and interleukin-13 (IL-13) signaling and has demonstrated efficacy in chronic pruritic disorders such as atopic dermatitis, chronic spontaneous urticaria, and prurigo nodularis. This supports a potential role of dupilumab in the management of uremic pruritus and ARPC as observed in our patient.^1^^,^^4^

## Case report

A 71-year-old female presented to the dermatology department with a 4-m history of severe generalized pruritus associated with multiple keratotic papules, nodules, and excoriation marks over the back and extremities. Pruritus significantly disrupted her sleep and daily activities. The patient is a known case of lower-limb ischemia, hypertension, and a 35-y history of type 2 diabetes mellitus complicated by diabetic retinopathy and stage 3b–A2 chronic kidney disease.

**Fig 1:**
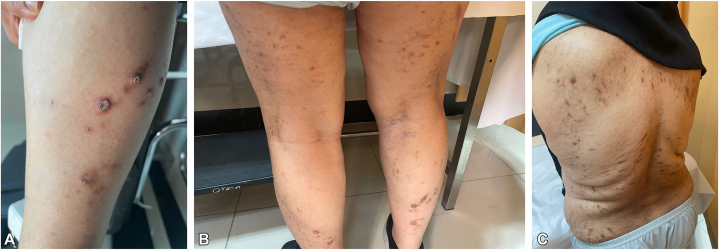
Multiple excoriated papules and nodules with central crust on both legs **(A, B)**. Postinflammatory hyperpigmentation **(C)**.

Physical examination revealed numerous well-demarcated brown, umbilicated papules and nodules with erythematous rims and central keratotic plugs. Lesions were symmetrically distributed over the trunk and limbs and were associated with excoriation marks and postinflammatory hyperpigmented macules (Fig 1). Our differential diagnoses included acquired perforating dermatosis, prurigo nodularis, and arthropod bite reaction.

**Fig 2:**
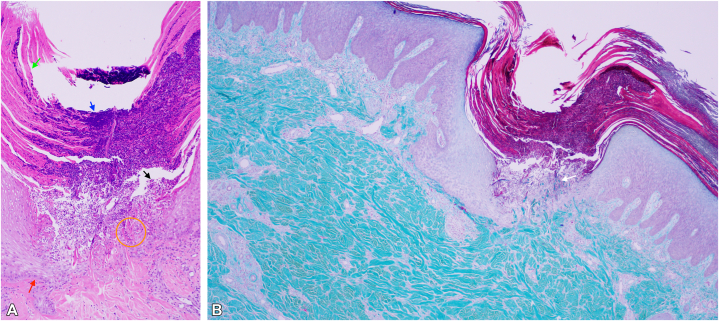
Cup-shaped invagination composed of keratin (*green arrow*), inflammatory infiltrate (*black arrow*), and basophilic debris (*blue arrow*). The underlying epidermis was thinned (*red arrow*), with transepidermal elimination of vertically oriented degenerated collagen fibers (*orange circle*) **(A)**. Transepidermal elimination of collagen fibers stained blue by Masson’s trichrome **(B)**.

A 4-mm skin punch biopsy was obtained from the right leg. Histopathologic examination showed mild acanthosis with transepidermal elimination of collagen and superficial perivascular inflammatory infiltrates (Fig 2). These findings were consistent with a diagnosis of ARPC in the setting of UP. Laboratory investigations were notable for renal dysfunction and poor glycemic control. Serum creatinine was 125 μmol/L (reference range, 44-80), urea 7.9 mmol/L (2.86-7.14), estimated glomerular filtration rate 38 mL/min/1.73 m^2^ (90-140), HbA1c 9.4% (4-5.6), urine creatinine 5.5 mmol/L (7-15.8), and urine albumin-to-creatinine ratio was 3.7 mg/mmol (0.1-3).

The patient was treated with oral antihistamines, topical potent corticosteroids, and emollients for 7 m with minimal improvement. She refused narrowband UVB phototherapy because of the difficulty in attending hospital visits 2 to 3 times weekly. Systemic corticosteroids were not advised by her primary physician due to uncontrolled diabetes and diabetic nephropathy.

**Fig 3:**
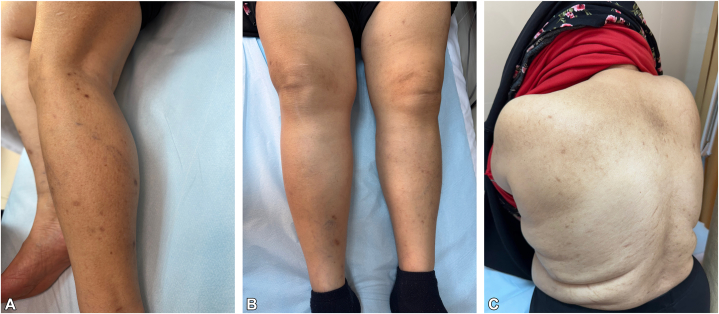
Clinical pictures of the patient at 6 wk **(A)** and 10 m **(B, C)** after dupilumab treatment.

After obtaining informed consent, dupilumab monotherapy was initiated at a loading dose of 600 mg, followed by 300 mg every 2 weeks. The patient reported significant improvement in quality of life 4 weeks after treatment initiation. At 6 months, her skin lesions healed completely, leaving only postinflammatory hyperpigmentation (Fig 3). Her pruritus Numeric Rating Scale score decreased from 10 to 0. During the 10-month follow-up period, she continued dupilumab monotherapy without any reported adverse effects.

## Discussion

ARPC is an uncommon perforating dermatosis characterized by transepidermal elimination of altered collagen fibers. The pathogenesis of ARPC is not fully understood. Repetitive scratching is believed to promote collagen degeneration, and concomitant microangiopathy may impair the clearance of metabolic byproducts.^1^ ARPC is most frequently associated with chronic kidney disease, followed by diabetes mellitus.^1^^,^^3^

Previous studies have demonstrated that UP affects up to 84% of patients with end-stage renal disease and impairs their quality of life.^4^^,^^5^ Current treatment options for UP include gabapentin, cromolyn sodium, antihistamines, antidepressants, neuroleptics, and phototherapy. These medications have variable efficacy and can be associated with adverse effects, particularly in elderly patients with multiple comorbidities.^5^

Growing evidence suggests that type 2 immune dysregulation plays a role in the pathogenesis of ARPC and uremic pruritus. It has been found that patients with UP have increased serum levels of IL-31, which is a key pruritogenic cytokine that is produced primarily by Th2 cells.^6^^,^^7^ Recent studies on ARPC have reported increased dermal infiltration of CD3^+^ T cells and a predominance of Th2 cells, resembling the patterns observed in atopic dermatitis. Furthermore, IL-4 and IL-13 cytokines, which promote itch, are upregulated in ARPC.^1^

IL-4 is the key driver of Th2 differentiation, proliferation, and subsequent production of IL-4, IL-13, and IL-31. IL-4 and IL-13 upregulate the expression of interleukin-31 receptor α (IL-31Rα) on cutaneous sensory neurons and increase their sensitivity to multiple pruritogens, including IL-31 and histamine. The subsequent binding of IL-4, IL-13, and IL-31 to their receptors on sensory neurons results in neuronal activation and promotes chronic itch.^7^ Dupilumab is a fully human monoclonal antibody that binds to the IL-4 receptor alpha subunit, thus inhibiting both IL-4 and IL-13 signaling pathways. It has also been demonstrated that dupilumab downregulates the expression of IL-31 and IL-31 receptors in atopic dermatitis lesional skin.^7^^,^^8^ These mechanisms support its use as an antipruritic agent in perforating dermatoses and UP.^3^, ^4^, ^5^^,^^9^^,^^10^ In addition, dupilumab is approved for the treatment of several inflammatory dermatoses, including atopic dermatitis, chronic spontaneous urticaria, bullous pemphigoid, and prurigo nodularis.^2^ It has also demonstrated antipruritic effects in other conditions such as lichen planus, malignancy-associated pruritus, and chronic pruritus of unknown origin.^9^^,^^10^

## Conclusion

The management of ARPC and UP remains challenging, particularly in elderly patients with multiple comorbidities. In our case, dupilumab monotherapy results in marked improvement in pruritus and skin lesions; however, larger studies are needed to confirm its efficacy and safety.

## Conflicts of interest

None disclosed.
